# Report of the Surgical Clinic of the Atlanta Medical College

**Published:** 1855-09

**Authors:** W. F. Westmoreland

**Affiliations:** Professor of Surgery


ARTICLE VII.—*Report of the Surgical Clinic of the Atlanta Medical College.* By W. F. WESTMORELAND, M. D., Professor of Surgery.

In this article we propose merely to commence a report, to be continued in the next number of this Journal, of some of the more interesting cases operated on and prescribed for before the first class of the Atlanta Medical College.

CASE 1—*Stone—Lateral Operation.*—Henry, a boy, aged six years, was placed under my care early in May by Dr. Iverson, of Fayette County.

The boy had been laboring under all the symptoms of stone in the bladder for two or three years previous to my seeing him. His sufferings at each paroxysm were intense; but notwithstanding the duration and severity of the symptoms, he was in most excellent health.

Upon his arrival in Atlanta, all the symptoms were greatly aggravated, he having made fifteen miles of the trip on horseback. All subsided, however, at the expiration of a few days.

The day after his arrival the sound was introduced, and a stone readily detected, which was thought to be quite large for one of his age.

The day previous to the operation a dose of the Sulphate of Magnesia was administered, and immediately preceding the operation a half pint of water was thrown up the bowels to remove any fecal matter that might have lodged in the rectum—this being all the preparation I think necessary when the patient is in good health. Certainly, if the patient be greatly debilitated, we may attempt, by an appropriate course of treatment, to give some vigor to the constitution before performing the operation; but as this state of the system is generally dependent upon the irritation and pain set up by the presence of the stone, we seldom accomplish much; yet, under some circumstances, it is necessary to attempt it.

After bringing the patient under the influence of chloroform, the lateral operation, with the single *Lithotome Cache* was performed in the usual way. The stone was seized with the forceps, and although of considerable size, (but not so large as we at first imagined,) was readily extracted.

The calculus was composed of the Phosphate of Lime; measured four and a half inches in one circumference and two and a half in the other. Weight not recollected.

There was rather more hemorrhage immediately after the operation than is usual, but it was readily arrested by injecting a stream of cold water into the wound. The patient did well the first three days, but on the morning of the fourth there was secondary hemorrhage to a considerable extent; lasted, however, but a very short time; ceased before I reached the patient, and in all lost perhaps three or four ounces of blood. There was no return of hemorrhage, nor was there any unpleasant symptom. Upon the eighth day the urine assumed its natural channel.

I should have remarked that the urine passed, to some extent, per urethra previous to the hemorrhage above alluded to; but after the hemorrhage, and until the eighth day after the operation, the urine passed alone by the incision in the perineum.

The patient was discharged the fifteenth day after the operation.

CASE 2.—*Syphilis, Section of the Tendo Achillis.*—David, a servant of the Rev. Mr. B., of Hancock County, entered my Infirmary for treatment some time in May. David is a mulatto, between 50 and 55 years of age, and at one time certainly had a most excellent constitution.

The following is the history obtained from the patient at my first examination. Three years ago he was attacked with sore throat, which yielded to a few applications of the Nitrate of Silver.

About the time this affection of the throat subsided, or before it had entirely disappeared, there appeared upon the forehead and face an eruption, which his physician decided was syphilitic. Under an appropriate treatment, mercury being the basis, I suppose, as he says his teeth were very sore, the eruption in a few weeks entirely disappeared.

Four or five months after the treatment for the above eruption was suspended, there appeared upon the extremities another form of eruption, which, from his description, as well as the manner in which the ulcers cicatrized, was syphilitic rupia.

He says that the ulcers were the result of the softening of small tumors, situated under the skin, they feeling when caught up between the thumb and fingers like a bullet or marble beneath the cuticle.

There appears to have been a group of these tumors upon the left leg, situated over the gastrocnemius muscle, between it and the solens, and extending down to near the ankle joint. There resulted from this, he says, an extensive ulcerating surface. I am thus particular in describing the ulcers or tumors in this region, as they account for the great contraction of the tendo achillis, which existed when I first saw the patient.

Of the treatment pursued when in the above situation he knows nothing. Passed through the hands of two or three physicians before the ulcers were disposed to cicatrize.

He positively denies ever having had a chancre upon the genital organs. The physician who first attended him did not succeed in finding the trace of a chancre.

The following was the appearance presented when I first saw him. There were upon the legs, arms, and trunk large cicatrises, it being evident, from their appearance, that the cuticle at those points had been entirely destroyed. Immediately over the belly of the gastrocnemius there was a large and ugly cicatrix, this muscle not being more than one-third the size of its fellow upon the opposite leg. Just above the internal malleolus there commenced an ulcer which extended obliquely across the leg, its centre being about the point where the gastrocnemius becomes tendinous. The tendo achillis was greatly contracted, drawing the heel up as in Talipes Equinus.

I should have remarked that I prescribed for the patient two or three months before seeing him. I gave him the Syrup of Gentian and the Iodide of Potassium, dressing the ulcer with Calomel. He says that this reduced greatly the size of the ulcer. This prescription was continued, and in three weeks the ulcer was half the size it was when he entered the Infirmary.

A section was now made of the tendo achillis, and the foot and leg placed in an apparatus to bring down the heel. In ten days the foot was at a right angle with the leg, and in two months from his entrance into the hospital the ulcer had cicatrized, and he able to walk without his crutches. He is now at work and doing well. He still continues the Iodide of Potassum in small doses.

The above, in my opinion, is beyond all doubt a case of syphilis; still I feel pretty sure that there was never a chancre upon the genital organs.

For those who believe that secondary syphilis is contagious, it would, without further investigation, be a case in favor of their views. But as I am convinced that Ricord is correct upon this subject—that secondary syphilis cannot be communicated except by transmission from parent to child—I shall proceed to account in some other way for the origin of this case.

The patient at the time, and for some weeks before, the development of the disease, had worked with a boy who, he says, had a chancre upon the genital organs. Both being mechanics, and consequently handling the same tools, it occurred to me that the disease might have been communicated in this way. I asked him in this connection (without his being at all conscious of the object of the question) if he had a sore upon the hand when he was at work with this boy. He said he had a sore upon the finger from striking it against a plank, a month or more before he had the sore throat, which lasted three or four weeks. He showed me the cicatrix upon the finger, which is still very well marked.

My impression is, that after wounding the finger with the plank, in handling the tools that the other boy had been using, or in some other way, this wound was inoculated with the virus from the chancre upon the other boy.

